# Ipsilesional Handgrip Strength and Functional Outcomes After Stroke: A Retrospective Cohort Study

**DOI:** 10.3390/jcm15155925

**Published:** 2026-07-29

**Authors:** Eidmantė Lileikyte, Karolina Lukaševič, Raimondas Savickas, Laura Petrusevičienė

**Affiliations:** Department of Rehabilitation, Lithuanian University of Health Sciences, 44307 Kaunas, Lithuania; eidmante.lileikyte@stud.lsmu.lt (E.L.); karolina.lukasevic@stud.lsmu.lt (K.L.); raimondas.savickas@lsmu.lt (R.S.)

**Keywords:** handgrip strength, stroke rehabilitation, contralesional hand, ipsilesional hand, neurorehabilitation

## Abstract

**Background and Objectives***:* Handgrip strength of the ipsilesional hand is increasingly recognized as a prognostic marker in stroke rehabilitation. Therefore, this retrospective observational cohort study investigated the association between baseline ipsilesional handgrip strength and functional outcomes after subacute stroke, while additionally incorporating age- and sex-adjusted normative handgrip strength thresholds, sex-stratified analyses, and exploratory regression models. **Materials and Methods***:* This study was conducted on 180 ischaemic stroke patients admitted to the Neurorehabilitation Unit between 2020 and 2024. Handgrip strength of both hands was measured at admission and discharge. Functional status was assessed using standardized assessments: the Functional Independence Measure (FIM) and the Barthel Index (BI). Correlation analyses were performed to evaluate associations between handgrip strength and functional outcomes. **Results***:* Among 180 participants, those with age- and sex-adjusted normative ipsilesional handgrip strength demonstrated significantly higher FIM scores both before and after rehabilitation (*p* = 0.008 and *p* = 0.042), whereas no significant differences were found for the remaining functional outcome measures. In sex-stratified regression analyses, baseline handgrip strength of the ipsilesional hand was significantly associated with FIM score at discharge in both male (B = 0.705, 95% CI: 0.312 to 1.099, *p* < 0.001) and female participants (B = 0.735, 95% CI: 0.076 to 1.394, *p* = 0.029). **Conclusions***:* The results of this study confirmed that handgrip strength of the ipsilesional hand is significantly associated with functional outcomes at the end of inpatient stroke rehabilitation. These findings support its potential value as a simple clinical indicator of functional recovery, although the proposed regression models require external validation before clinical application.

## 1. Introduction

Stroke remains a prevalent neurological condition worldwide, associated with significant mortality and long-term functional impairments, such as motor and cognitive deficits, speech disorders, and reduced mobility [1,2,3]. Although stroke primarily results in motor deficits on the contralesional side—commonly referred to as the affected side—emerging evidence highlights the clinical significance of impairments in the ipsilesional limbs, which are traditionally considered unaffected. Previous studies have demonstrated that up to 37% of stroke survivors experience motor dysfunction in the ipsilesional upper limb [4,5]. Therefore, the new evidence suggests that both sides are affected after a unilateral stroke, and the ipsilesional side should be considered as less affected rather than unaffected.

This phenomenon can be partially explained by the anatomical structure of the corticospinal tract, as about 10–15% of its fibers descend ipsilaterally without crossing over, which contributes to motor deficits on the ipsilateral side [6]. The decussating part is called the lateral corticospinal tract (LCT), which predominantly innervates distal extremity muscles on the contralateral side. In contrast, the anterior corticospinal tract (ACT) projects bilaterally and primarily innervates axial muscles, such as those of the trunk, neck, and shoulders. While the anterior corticospinal tract contributes to general motor control and does not directly affect handgrip strength, damage to ACT can substantially reduce handgrip strength due to impaired activation of trunk and shoulder muscles [7,8,9].

In addition to the corticospinal tract, the reticulospinal tract may also play a compensatory role in motor control following stroke. Originating from the brainstem reticular formation, the reticulospinal tract primarily descends ipsilaterally with limited crossover and is mostly involved in postural control, locomotion, and proximal limb movements. Studies show that in cases of severe damage to the corticospinal tract, functional enhancement of the reticulospinal tract can help restore gross motor function. However, this compensation is often achieved at the expense of fine motor control and may contribute to increased muscle tone and spasticity [10,11]. In addition, the anatomical characteristics of the reticulospinal tract may also play a part in ipsilesional motor deficits after stroke and may impact arm strength, specifically during tasks requiring force or posture [11]. Handgrip strength, commonly assessed via dynamometry, has emerged as a simple yet reliable indicator of upper-limb function. Beyond reflecting hand-specific performance, handgrip strength serves as a proxy for overall neuromuscular coordination and is linked to proximal muscle activity, particularly in the shoulder girdle. The rotator cuff muscles—supraspinatus, infraspinatus, subscapularis, and teres minor—play a central role in shoulder stabilization and upper-limb coordination. Increased shoulder strength, particularly in the rotator cuff region, is positively associated with improved arm strength, most likely due to coordinated proximal stabilization and distal function during grasping tasks [12,13,14].

Importantly, handgrip strength is also associated with broader systemic conditions such as frailty and sarcopenia—both of which are increasingly recognized as independent predictors of poor functional outcomes after stroke [3]. Frailty, characterized by decreased physiological reserve and increased vulnerability to stressors, is linked to delayed recovery and greater disability [3]. Sarcopenia, described as a gradual loss of skeletal muscle mass and strength, can lead to further deterioration in motor function and rehabilitation potential [15]. Since handgrip strength is a fundamental factor in assessing both conditions, its measurement can provide valuable predictive information that extends beyond localized limb function [16].

A comprehensive assessment of functional abilities at the onset of rehabilitation and before discharge from the hospital is typically performed using the Barthel Index (BI) and the Functional Independence Measure (FIM). The BI evaluates independence in essential activities of daily living, such as feeding, bathing, dressing, toileting, and mobility, providing an overview of the patient’s basic self-care abilities. In contrast, the FIM offers a broader assessment covering both motor and cognitive domains, including communication, social interaction, and decision-making. An early assessment using BI and FIM establishes a baseline level of functional independence, helps to create an individual rehabilitation plan, and allows progress to be monitored over time. Clinically, handgrip strength evaluation is a simple, low-cost and minimally training-requiring procedure that can be used as a practical tool in both acute and rehabilitation periods. The integration of hand strength measurement with BI and FIM yields a more comprehensive assessment of functional ability and improved prediction of rehabilitation outcome [17,18].

During stroke rehabilitation, clinical focus is typically directed toward the more affected contralesional side. However, when recovery of this limb is limited due to a large ischaemic area, the ipsilesional limb plays a crucial role in compensating for performing activities of daily living. Therefore, impairment in the ipsilesional limb further complicates the functional independence of stroke survivors [4]. Even though the prognostic significance of ipsilesional handgrip strength in the early subacute stroke phase is already established in previous studies, this retrospective observational cohort study additionally incorporates age- and sex-adjusted normative handgrip strength thresholds, applies sex-stratified analyses, and develops clinically applicable prediction equations for functional outcomes.

The aim of this retrospective observational cohort study was to investigate the relationship between baseline ipsilesional handgrip strength and functional recovery, assessed using FIM and BI in patients with subacute ischaemic stroke. In addition, age- and sex-adjusted normative handgrip strength thresholds were applied, sex-stratified analyses were performed, and exploratory regression models were developed to further characterize the association between ipsilesional handgrip strength and functional outcomes.

## 2. Materials and Methods

### 2.1. Participants

This study was designed as a retrospective observational cohort study conducted at the Hospital of Lithuanian University of Health Sciences Kaunas Clinics between January 2020 and December 2024. The study was conducted in accordance with the Declaration of Helsinki and approved by the Bioethics Centre of the Lithuanian University of Health Sciences (protocol code 2025-BEC2-0404; date of 19 March 2025). During this period, a total of 295 patients with first-ever ischaemic stroke were admitted to the Neurorehabilitation Unit. Patients were assigned to a seven-week inpatient rehabilitation program, which consisted of daily individualized physiotherapy and occupational therapy sessions, performed by qualified specialists, as well as functional electrical stimulation (FES), massages, speech therapy, and consultations with a psychologist and a social worker.

**Figure 1:**
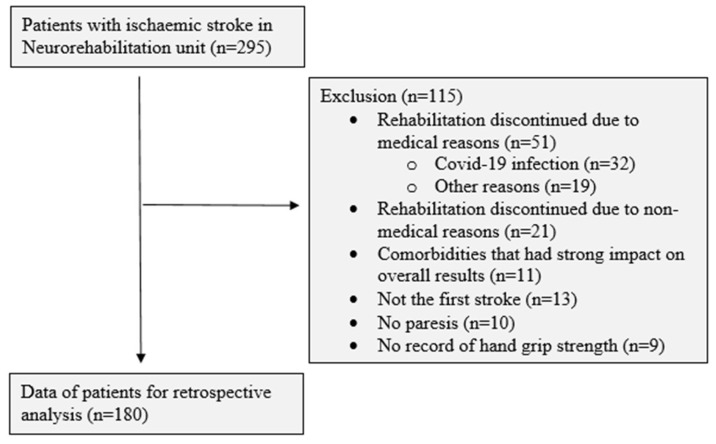
Study flow chart. *n*—number of cases.

Patient eligibility criteria included adults (≥18 years old) who had experienced a first-ever ischaemic stroke and did not have severe comorbid conditions that could affect handgrip strength. Patients were excluded from the study if they discontinued rehabilitation due to both medical and non-medical reasons, had significant comorbidities, a history of prior stroke, absence of motor deficits, or missing handgrip strength data. After applying the inclusion and exclusion criteria, a total of 180 patients were included in the final analysis. The participant selection process is presented in Figure 1.

### 2.2. Data Collection

Demographic and clinical data extracted from patient medical records included the following: age; sex; time from stroke onset to rehabilitation admission; lesion location (right hemisphere, left hemisphere, or vertebrobasilar system); side of motor impairment; stroke treatment method (intravenous thrombolysis [IVT], mechanical thrombectomy [MTE], both, or none); the presence of hemorrhagic transformation; speech impairments (motor aphasia, sensorimotor aphasia, dysarthria, or no impairment); handgrip strength of both ipsilesional and contralesional hands; FIM score and BI score on admission and at discharge.

The contralesional hand was defined as the upper extremity contralateral to the cerebral lesion and clinically corresponding to the more affected hand, whereas the ipsilesional hand was referred to the upper extremity ipsilateral to the lesion and clinically corresponding to the less affected hand. Stroke hemisphere, lesion laterality, and the affected side were determined from the neurological examination findings and neuroimaging (CT and/or MRI) documented in the medical records.

### 2.3. Handgrip Strength Measurement

Handgrip strength was measured using a hydraulic hand dynamometer (SH5001; SAEHAN Corporation, Changwon, Republic of Korea). Participants were assessed in a seated position with the shoulder adducted, elbow flexed to 90°, and the wrist in a neutral position, following standardized handgrip strength assessment procedures [19]. The ipsilesional hand was assessed first, followed by the contralesional hand. Participants performed three maximal voluntary contractions with each hand, separated by a 30-s rest interval, and the highest value was used for analysis. All measurements were performed by trained occupational therapists on admission and at discharge. The change in handgrip strength (Δ Handgrip strength) was calculated as the difference between discharge and admission values.

Normative handgrip strength was determined according to age- and sex-specific reference values reported by Tomkinson et al., derived from an international systematic review and individual participant data meta-analysis including healthy adults from multiple countries [20]. Handgrip strength at or above the 20th percentile (P20) was classified as normative.

### 2.4. Functional Outcome Measures

Functional status was evaluated on admission and at discharge using two standardized scales: FIM [21] and BI [22]. Changes in functional scores were calculated (∆FIM and ∆BI) to assess functional improvement during the rehabilitation stay.

### 2.5. Statistical Analysis

Statistical analyses were conducted using IBM SPSS Statistics 30.0 (IBM Corp., Armonk, NY, USA). Data are presented as numbers of cases (*n*), percentages (%), medians, and minimum–maximum values. Normality was assessed using the Kolmogorov–Smirnov test. As the data were non-normally distributed, comparisons between two independent groups were conducted using the Mann–Whitney U test, while paired comparisons were performed using the Wilcoxon signed-rank test. Associations between age, handgrip strength (ipsilesional and contralesional hands), and functional outcomes (FIM and BI at discharge, ∆FIM, ∆BI) were evaluated using Spearman’s rank correlation (ρ). Analyses were performed for the total cohort and additionally stratified by sex and by age groups (≤59 and ≥60 years). Sex-stratified linear regression analyses were performed to examine whether baseline handgrip strength of the ipsilesional hand predicts functional outcomes at discharge. Statistical significance was set at *p* < 0.05.

The study was powered to detect moderate correlations between baseline handgrip strength and functional outcomes. A sample size calculation using G*Power (version 3.1.9.7, two-tailed test, α = 0.05) indicated that detecting a moderate effect size (r = 0.30) requires 84 participants for 80% power and 112 participants for 90% power. The final sample exceeded the required sample size and ensured adequate statistical power for detecting moderate associations.

## 3. Results

### 3.1. Demographic and Clinical Characteristics

A total of 295 patients with ischaemic stroke were admitted to the Neurorehabilitation Unit between 2020 and 2024. After applying the exclusion criteria, data of 180 patients were included in the final analysis.

Ninety-six patients (53.3%) were male, and 84 (46.7%) were female. The overall median age was 70 years (range 23–95), with female participants being significantly older than males (73.0 years (27.0–91.0) vs. 65.0 years (23.0–95.0), *p* < 0.001). Demographic and clinical characteristics of the study population at the beginning of rehabilitation are summarized in Table 1.

Age- and sex-adjusted normative ipsilesional handgrip strength (≥P20) was observed in 100 participants (55.6%), whereas 80 participants (44.4%) demonstrated handgrip strength below the P20 threshold. Participants with normative ipsilesional handgrip strength had significantly higher FIM scores both before and after rehabilitation (*p* = 0.008 and *p* = 0.042), whereas no significant differences were found for the remaining functional outcome measures. Detailed results are presented in Table 2.

Median handgrip strength of the ipsilesional hand for male participants was 36.0 (range 10.0–70.0) before rehabilitation. After rehabilitation, it significantly increased to 41.4 (10.0–70.0) with a median change of 4.0 (0.0–24.0; *p* < 0.001). For female participants, the median handgrip strength of the ipsilesional hand before and after rehabilitation was 18.0 (2.0–40.0) and 22.0 (12.0–41.0), with a significant median change of 4.0 (−2.0–24.0; *p* < 0.001). The median change in handgrip strength of the ipsilesional hand did not differ between male and female participants (*p* = 0.987).

In the total cohort, the median FIM score increased from 38.0 (11.0–88.0) before rehabilitation to 75.5 (25.0–114.0) after rehabilitation (*p* < 0.001), with a median change of 32.0 (2.0–75.0). The median BI score increased from 15.0 (10.0–35.0) before rehabilitation to 50.0 (15.0–80.0) after rehabilitation (*p* < 0.001), with a median change of 30.0 (5.0–65.0).

In the total cohort, higher age was negatively correlated with all functional outcome measures (FIM and BI score at the end of rehabilitation, as well as changes in FIM (∆FIM) and BI (∆BI) during rehabilitation). Both hands’ handgrip strength before rehabilitation showed positive correlations with all functional outcome measures. The Strongest correlation was observed between handgrip strength of the ipsilesional hand before rehabilitation and FIM score at the end of rehabilitation, and handgrip strength of the contralesional hand before rehabilitation and BI at the end of rehabilitation, as well as ∆BI. Detailed results of the relationship between age and both hands’ handgrip strength before rehabilitation and functional outcomes are presented in Table 3.

### 3.2. Sex-Based Differences in Handgrip Strength and Functional Outcomes

In male participants, baseline handgrip strength of the ipsilesional hand was positively correlated with all functional outcome measures, including FIM and BI scores after rehabilitation, as well as ∆FIM and ∆BI. Handgrip strength of the contralesional hand showed positive correlations with post-rehabilitation FIM and BI scores and with ∆BI, whereas its association with ∆FIM was not significant.

In female participants, baseline handgrip strength of the ipsilesional hand was positively correlated with FIM score at discharge, but showed no significant associations with BI score after rehabilitation or with ∆FIM and ∆BI. Handgrip strength of the contralesional hand was positively correlated with post-rehabilitation FIM and BI scores and with ∆BI, while its correlation with ∆FIM was not significant.

**Figure 2:**
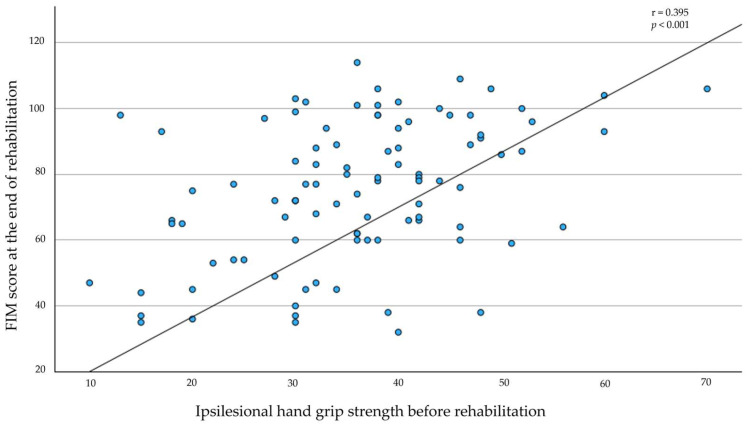
Association between ipsilesional handgrip strength before rehabilitation and FIM score at discharge in male participants.

**Figure 3:**
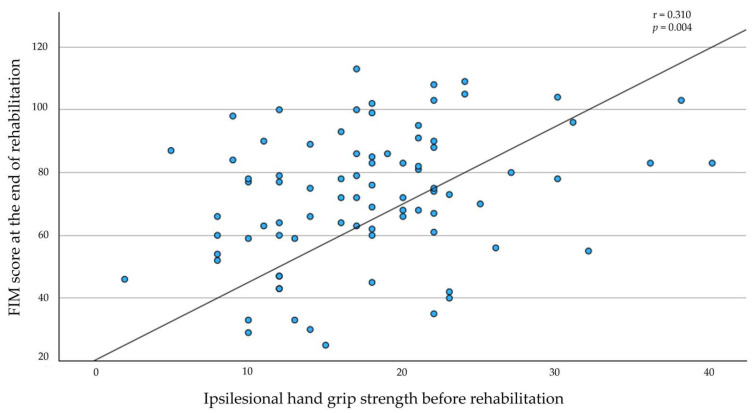
Association between ipsilesional handgrip strength before rehabilitation and FIM score at discharge in female participants.

All correlation coefficients and *p* values are presented in Table 4. Representative scatter plots illustrating associations between ipsilesional handgrip strength before rehabilitation and FIM score at discharge in men and women are shown in Figure 2 and Figure 3, respectively.

### 3.3. Age-Related Differences in Handgrip Strength and Functional Outcomes

In patients younger than 60 years, ipsilesional handgrip strength before rehabilitation showed a positive correlation with FIM at discharge (r = 0.313, *p* = 0.036), while associations with BI at discharge (r = 0.266, *p* = 0.078), ∆FIM (r = 0.193, *p* = 0.204), and ∆BI (r = 0.231, *p* = 0.127) were not significant. In patients older than 60 years, ipsilesional handgrip strength also showed a positive correlation with FIM at discharge (r = 0.24, *p* = 0.005), and had no significant correlations with BI at discharge (r = 0.131, *p* = 0.130), ∆FIM (r = 0.081, *p* = 0.351), or ∆BI (r = 0.105, *p* = 0.223).

In patients younger than 60 years, contralesional handgrip strength before rehabilitation showed positive correlation with FIM (r = 0.537, *p* < 0.001) and BI at discharge (r = 0.623, *p* < 0.001), as well as ∆ BI (r = 0.624, *p* < 0.001), but had no significant correlation with ∆ FIM (r = 0.236, *p* < 0.118). In patients older than 60 years, contralesional handgrip strength before rehabilitation positively correlated with all functional outcomes: FIM at discharge (r = 0.392, *p* < 0.001), BI at discharge (r = 0.350, *p* < 0.001), ∆FIM (r = 0.185, *p* = 0.032), and ∆BI (r = 0.313, *p* < 0.001).

Detailed correlation analyses stratified by age group are provided in Appendix A (Table A1 and Table A2).

### 3.4. Prediction Models for Functional Outcomes

In male participants, baseline handgrip strength of the ipsilesional hand was a significant predictor of FIM score at discharge (B = 0.705, 95% CI: 0.312 to 1.099, *p* < 0.001). This model explained 18.6% of the variance in FIM outcomes (R^2^ = 0.186). The resulting prediction equation was:

$${\mathrm{FIM}}_{\mathrm{after}}=62.252+0.705\times {Handgrip\;Strength}_{Ipsilesional}$$

In female participants, baseline handgrip strength of the ipsilesional hand significantly predicted FIM score at discharge (B = 0.735, 95% CI: 0.076 to 1.394, *p* = 0.029). The model explained 12.8% of the variance in FIM outcomes (R^2^ = 0.128). The resulting simplified prediction equation was:

$${\mathrm{FIM}}_{\mathrm{after}}=78.985+0.735\times {Handgrip\;Strength}_{Ipsilesional}$$

Age was not a significant predictor for both male (*p* = 0.283) and female (*p* = 0.133) participants and was therefore excluded from the simplified formula.

In male participants, baseline handgrip strength of the ipsilesional hand significantly predicted BI score at discharge (B = 0.442, 95% CI: 0.080 to 0.805, *p* = 0.017), while age was also a significant negative predictor (B= –0.339, 95% CI: –0.670 to –0.009, *p* = 0.044). The model explained 16.0% of the variance in BI outcomes (R^2^ = 0.160). The resulting prediction equation was:

$${\mathrm{BI}}_{\mathrm{after}}=57.478-Age+0.442\times {Handgrip\;Strength}_{Ipsilesional}$$

In female participants, baseline handgrip strength of the ipsilesional hand did not significantly predict discharge BI score (B = 0.242, 95% CI: –0.383 to 0.867, *p* = 0.443). Age was also not a significant covariate (B = –0.276, 95% CI: –0.625 to 0.072, *p* = 0.119). The overall model was not statistically significant (*p* = 0.095) and explained only 5.6% of the variance (R^2^ = 0.056).

## 4. Discussion

The present study revealed that greater baseline ipsilesional handgrip strength was associated with better functional outcomes at discharge, as assessed by FIM and BI. The exploratory sex-stratified regression analyses also showed that baseline ipsilesional handgrip strength was significantly associated with FIM score at discharge in both male and female participants. Although these findings are consistent with previous studies reporting a prognostic association between ipsilesional handgrip strength and post-stroke recovery, our study extends the existing evidence by incorporating age- and sex-adjusted normative thresholds and sex-stratified analyses. These findings support the concept that handgrip strength of the ipsilesional hand is a simple, low-cost, and highly accessible prognostic marker of functional recovery after a unilateral stroke. Although significant associations were also observed between contralesional handgrip strength and functional outcomes, the present study primarily focuses on the clinical relevance of ipsilesional handgrip strength, as its prognostic value has been less well investigated. Importantly, the results showed that almost half of all participants demonstrated age- and sex-adjusted ipsilesional handgrip strength below the normative threshold at the beginning of rehabilitation. This finding supports previous evidence indicating that ipsilesional limb impairment is common following unilateral stroke and that the so-called ‘unaffected’ hand often exhibits measurable weakness and motor deficits [4,5,6]. The purpose of applying normative reference values was to determine whether ipsilesional handgrip strength was preserved relative to healthy individuals of the same age and sex; therefore, these findings suggest that clinically relevant impairment of the ipsilesional limb is present in a substantial proportion of stroke survivors.

Participants with normative ipsilesional handgrip strength demonstrated significantly better FIM scoring at the beginning of the rehabilitation and at discharge. However, there were no differences between groups in BI score at either time point, as well as in ∆ FIM and ∆BI. The absence of differences in changes in functional outcome measures during rehabilitation indicates that individuals with lower ipsilesional handgrip strength improved at a comparable rate to those with normative strength, although they may require a longer rehabilitation stay to achieve similar functional levels.

In male participants, the baseline ipsilesional handgrip strength showed significant correlations with all functional outcome measures. In contrast, among women, handgrip strength demonstrated a significant association only with FIM score at discharge, while its relationships with other functional indicators were not statistically significant. Several explanations may account for these sex-related differences. First, women typically have lower absolute handgrip strength and narrower strength distributions [23], which may reduce statistical sensitivity. Second, functional recovery in women after stroke is known to be more heterogeneous, potentially influenced by statistically higher age at stroke onset, higher rates of comorbidities, and post-stroke depression compared with men [23]. Finally, the FIM is considered to be more sensitive to functional assessment after severe stroke than the BI [18], allowing it to detect smaller changes in functional independence. Similarly, the exploratory regression analyses identified a significant association with FIM in both sexes, whereas the association with BI was observed only in male participants.

Interestingly, age did not significantly contribute to the exploratory regression models. This observation is consistent with evidence suggesting that muscular strength may serve as a more accurate indicator of physical capacity than biological age [24,25]. On the other hand, age negatively correlated with functional outcomes in the total cohort. Moreover, younger patients showed stronger associations between handgrip strength (especially in the contralesional hand) and functional outcomes. Although the mechanisms underlying these age-related differences were beyond the scope of our present study, previous research suggests that age-related differences in neuroplasticity, skeletal muscle quality, and neuromuscular function may contribute to functional recovery after stroke [26,27,28].

Functional recovery after stroke may be influenced by multiple factors, including age, initial stroke severity, lesion characteristics, history of cerebrovascular events, and various comorbidities [2,13]. Although the present study did not account for all of these variables, it contributes to the understanding of the clinical relevance of ipsilesional handgrip strength as an indicator of post-stroke functional outcomes. Clinically, handgrip strength is a practical and informative measure that reflects not only hand function but also overall neuromuscular and general health. It involves coordinated activity throughout the kinetic chain of the upper limb [12,13] and has been associated with frailty, sarcopenia, and cardiovascular health [3,29], all of which may influence functional recovery after stroke.

### Study Limitations

Several limitations of this study should be acknowledged. First, this was a retrospective study conducted at a single neurorehabilitation center, which limits causal interference and may reduce the generalizability of the findings. Only patients admitted for inpatient rehabilitation were included; therefore, selection bias cannot be excluded. Second, although the primary objective of this study was to investigate the association between ipsilesional handgrip strength and functional outcomes after stroke, several factors known to influence post-stroke recovery—including stroke severity, lesion characteristics and cognitive impairment—were not included in the analyses. As a result, the exploratory regression models should not be interpreted as comprehensive clinical prediction models. Although these proposed regression equations provide an initial estimate of the association between baseline handgrip strength and functional status at discharge, they have not been externally validated and should be interpreted with caution. Finally, hand dominance was not routinely documented; hence, in this retrospective study, we could not take into account its influence on the functional outcomes. Since the dominant hand is generally stronger and may play a greater role in performing daily activities, it may have influenced both ipsilesional handgrip strength and functional outcomes.

## 5. Conclusions

The results of this study suggest that baseline handgrip strength of the ipsilesional hand is associated with functional outcomes at the end of inpatient stroke rehabilitation and can serve as an indicator of functional recovery. Given that similar associations between handgrip strength and functional outcomes have been reported in earlier studies, our findings primarily provide confirmatory evidence supporting the association between ipsilesional handgrip strength and post-stroke functional outcomes. Further prospective multicenter studies are needed to determine whether ipsilesional handgrip strength provides additional prognostic information beyond established predictors of stroke recovery.

## Figures and Tables

**Table 1:** Demographic and clinical characteristics of the study population at the beginning of rehabilitation.

Variables	Value
Sex	
male/female	96/84
Age group; *n* (%)	
≤59 years old	45 (25.0)
≥60 years old	135 (75.0)
Lesion localization; *n* (%)	
Right hemisphere	64 (35.6)
Left hemisphere	90 (50.0)
Vertebrobasal pool	26 (14.4)
Treatment; *n* (%)	
IVT	50 (27.8)
MTE	40 (22.2)
IVT and MTE	25 (13.9)
No specialized treatment	65 (36.1)
Hemorrhagic transformation; *n* (%)	30 (16.7)
Affected side; *n* (%)	
Right side	105 (58.3)
Left side	75 (41.7)
Speech disorders; *n* (%)	
Sensory aphasia	2 (1.1)
Motor aphasia	21 (11.7)
Sensorimotor aphasia	59 (32.8)
Dysarthria	59 (32.8)
No disorders	37 (20.7)
Time from stroke onset to rehabilitation (days); median (min–max)	16.0 (6.0–67.0)
Ipsilesional handgrip strength; median (min–max)	25.0 (2.0–70.0)
Contralesional handgrip strength; median (min–max)	1.0 (0.0–41.0)

*n*—number of cases; %—percentage; IVT—intravenous thrombolysis; MTE—mechanical thrombectomy; median—median value; min—minimum value; max—maximum value.

**Table 2:** Comparison of functional outcomes between participants with normative (≥P20) and low (<P20) ipsilesional handgrip strength.

Functional Outcome Measure	≥P20 (*n* = 100); Median (Min–Max)	<P20 (*n* = 80); Median (Min–Max)	*p* Value
FIM before rehabilitation	42.0 (11.0–88.0)	35.0 (20.0–78.0)	0.008
BI before rehabilitation	15.0 (10.0–35.0)	15.0 (10.0–35.0)	0.293
FIM at the end of rehabilitation	78.5 (25.0–114.0)	72.0 (29.0–113.0)	0.042
BI at the end of rehabilitation	50.0 (15.0–80.0)	50.0 (15.0–80.0)	0.421
∆FIM	35.0 (4.0–75.0)	30.0 (2.0–71.0)	0.371
∆BI	32.5 (5.0–65.0)	30.0 (5.2–65.0)	0.500

*n*—number of cases; min—minimum value; max—maximum value; FIM—functional independence measure; BI—Barthel index; ∆—difference between discharge and admission values.

**Table 3:** Correlations of age and baseline ipsilesional and contralesional handgrip strength with functional outcomes.

Functional Outcome Measures	Age	Handgrip Strength of the Ipsilesional Hand Before Rehabilitation	Handgrip Strength of the Contralesional Hand Before Rehabilitation
FIM at the end of rehabilitation	r = −0.286 *p* < 0.001	r = 0.300 *p* <0.001	r = 0.109 *p* <0.001
∆FIM	r = −0.233 *p* = 0.002	r = 0.153 *p* = 0.040	r = 0.175 *p* = 0.019
BI at the end of rehabilitation	r = −0.292 *p* < 0.001	r = 0.210 *p* = 0.005	r = 0.393 *p* < 0.001
∆BI	r = −0.277 *p* < 0.001	r = 0.182 *p* = 0.015	r = 0.359 *p* < 0.001

FIM—functional independence measure; BI—Barthel index, ∆—difference between discharge and admission values; r—correlation coefficient; *p*—*p* value.

**Table 4:** Sex-stratified associations between both hands’ handgrip strength and functional outcomes.

Functional Outcome Measures	Handgrip Strength of the Ipsilesional Hand Before Rehabilitation		Handgrip Strength of the Contralesional Hand Before Rehabilitation	
	Male	Female	Male	Female
FIM at the end of rehabilitation	r = 0.395 *p* < 0.001	r = 0.310 *p* = 0.004	r = 0.438 *p* < 0.001	r = 0.395 *p* < 0.001
∆FIM	r = 0.221 *p* = 0.031	r = 0.186 *p* = 0.090	r = 0.180 *p* = 0.079	r = 0.195 *p* = 0.075
BI at the end of rehabilitation	r = 0.344 *p* < 0.001	r = 0.143 *p* = 0.196	r = 0.370 *p* < 0.001	r = 0.426 *p* < 0.001
∆BI	r = 0.290 *p* = 0.004	r = 0.113 *p* = 0.307	r = 0.332 *p* < 0.001	r = 0.386 *p* < 0.001

FIM—functional independence measure; BI—Barthel index, ∆—difference between discharge and admission values; r—correlation coefficient; *p*—*p* value.

## Data Availability

The data presented in this study are available on request from the corresponding author. The data are not publicly available due to privacy reasons.

## Abbreviations

The following abbreviations are used in this manuscript:

FIM	Functional Independence Measure
BI	Barthel Index
LCT	Lateral corticospinal tract
ACT	Anterior corticospinal tract
IVT	Intravenous thrombolysis
MTE	Mechanical thrombectomy

## Appendix A

**Table A1:** Correlation of both hands’ handgrip strength before rehabilitation with functional outcome measures in patients younger than 60 years.

	Handgrip Strength of the Ipsilesional Hand Before Rehabilitation	Handgrip Strength of the Contralesional Hand Before Rehabilitation
FIM at the end of rehabilitation	r = 0.313 *p* = 0.036	r = 0.537 *p* < 0.001
∆FIM	r = 0.193 *p* = 0.204	r = 0.236 *p* = 0.118
BI at the end of rehabilitation	r = 0.266 *p* = 0.078	r = 0.623 *p* < 0.001
∆BI	r = 0.231 *p* = 0.127	r = 0.624 *p* < 0.001

FIM—functional independence measure; BI—Barthel index, ∆—difference between discharge and admission values; r—correlation coefficient; *p*—*p* value.

**Table A2:** Correlation of both hands’ handgrip strength before rehabilitation with functional outcome measures in 60-year-old or older patients.

	Handgrip Strength of the Ipsilesional Hand Before Rehabilitation	Handgrip Strength of the Contralesional Hand Before Rehabilitation
FIM at the end of rehabilitation	r = 0.240 *p* = 0.005	r = 0.392 *p* < 0.001
∆FIM	r = 0.081 *p* = 0.351	r = 0.185 *p* = 0.032
BI at the end of rehabilitation	r = 0.131 *p* = 0.130	r = 0.350 *p* < 0.001
∆BI	r = 0.105 *p* = 0.223	r = 0.313 *p* < 0.001

FIM—functional independence measure; BI—Barthel index, ∆—difference between discharge and admission values; r—correlation coefficient; *p*—*p* value.

## References

[1] Li X., He Y., Wang D., Rezaei M.J. (2024). Stroke rehabilitation: From diagnosis to therapy. Front. Neurol..

[2] Cho T.H., Jeong Y.J., Lee H.J., Moon H.I. (2019). Manual function of the unaffected upper extremity can affect functional outcome after stroke. Int. J. Rehabil. Res..

[3] Carvalho D.B., Freitas S.M.S.F., Alencar F.A.D., Silva M.L., Alouche S.R. (2020). Performance of discrete, reciprocal, and cyclic movements of the ipsilesional upper limb in individuals after stroke. Exp. Brain Res..

[4] Smith D.B., Scott S.H., Semrau J.A., Dukelow S.P. (2023). Impairments of the ipsilesional upper-extremity in the first 6-months post-stroke. J. Neuroeng. Rehabil..

[5] Semrau J.A., Herter T.M., Kenzie J.M., Findlater S.E., Scott S.H., Dukelow S.P. (2017). Robotic Characterization of Ipsilesional Motor Function in Subacute Stroke. Neurorehabil. Neural Repair.

[6] Bustrén E.L., Sunnerhagen K.S., Alt Murphy M. (2017). Movement Kinematics of the Ipsilesional Upper Extremity in Persons with Moderate or Mild Stroke. Neurorehabil. Neural Repair.

[7] Javed K., Reddy V., Forshing L. (2023). Neuroanatomy, lateral corticospinal tract. StatPearls [Internet].

[8] Natali A.L., Reddy V., Bordoni B. (2023). Neuroanatomy, corticospinal cord tract. StatPearls [Internet].

[9] Van Wittenberghe I.C., Peterson D.C. (2023). Corticospinal tract lesion. StatPearls [Internet].

[10] Akalu Y., Frazer A.K., Howatson G., Pearce A.J., Siddique U., Rostami M., Tallent J., Kidgell D.J. (2023). Identifying the role of the reticulospinal tract for strength and motor recovery: A scoping review of nonhuman and human studies. Physiol. Rep..

[11] Choudhury S., Shobhana A., Singh R., Sen D., Anand S.S., Shubham S., Baker M.R., Kumar H., Baker S.N. (2019). The Relationship Between Enhanced Reticulospinal Outflow and Upper Limb Function in Chronic Stroke Patients. Neurorehabil. Neural Repair..

[12] Pawar M., Ekbote V. (2024). Correlation Between Grip Strength and Scapular Muscles Strength Among Goldsmith Workers in Pune: A Correlational Study. Int. J. Health Sci. Res..

[13] Turabi R., Horsely I., Birch H., Jaggi A. (2022). Does grip strength correlate with rotator cuff strength in patients with atraumatic shoulder instability?. Bull. Fac. Phys. Ther..

[14] Olczak A. (2021). Motor coordination and grip strength in stroke patients: An observational study. Eur. J. Phys. Rehabil. Med..

[15] Ardeljan A.D., Hurezeanu R. (2023). Sarcopenia. StatPearls [Internet].

[16] Bohannon R.W. (2019). Grip strength: An indispensable biomarker for older adults. Clin. Interv. Aging..

[17] Winstein C.J., Stein J., Ross A., Bates B., Cherney L.R., Cramer S.C., Deruyter F., Eng J.J., Fisher B., Harvey R.L. (2016). Guidelines for adult stroke rehabilitation and recovery: A guideline for healthcare professionals from the American Heart Association/American Stroke Association. Stroke.

[18] Lee E.Y., Sohn M.K., Lee J.M., Kim D.Y., Shin Y.I., Oh G.J., Lee Y.S., Lee S.Y., Song M.K., Han J.H. (2022). Changes in Long-Term Functional Independence in Patients with Moderate and Severe Ischemic Stroke: Comparison of the Responsiveness of the Modified Barthel Index and the Functional Independence Measure. Int. J. Environ. Res. Public Health.

[19] Roberts H.C., Denison H.J., Martin H.J., Patel H.P., Syddall H., Cooper C., Sayer A.A. (2011). A review of the measurement of grip strength in clinical and epidemiological studies: Towards a standardised approach. Age Ageing.

[20] Tomkinson G.R., Lang J.J., Rubín L., McGrath R., Gower B., Boyle T., Klug M.G., Mayhew A.J., Blake H.T., Ortega F.B. (2025). International norms for adult handgrip strength: A systematic review of data on 2.4 million adults aged 20 to 100+ years from 69 countries and regions. J. Sport Health Sci..

[21] Keith R.A., Granger C.V., Hamilton B.B., Sherwin F.S. (1987). The functional independence measure: A new tool for rehabilitation. Adv. Clin. Rehabil..

[22] Mahoney F.I., Barthel D.W. (1965). Functional evaluation: The Barthel Index. Md. State Med. J..

[23] Puh U. (2010). Age-related and sex-related differences in hand and pinch grip strength in adults. Int. J. Rehabil. Res..

[24] Souza Saraiva W., Prestes J., Schwerz Funghetto S., Navalta J.W., Tibana R.A., da Cunha Nascimento D. (2019). Relation Between Relative Handgrip Strength, Chronological Age and Physiological Age with Lower Functional Capacity in Older Women. Open Access J. Sports Med..

[25] Riviati N., Indra B. (2023). Relationship between muscle mass and muscle strength with physical performance in older adults: A systematic review. SAGE Open Med..

[26] Sions J.M., Tyrell C.M., Knarr B.A., Jancosko A., Binder-Macleod S.A. (2012). Age- and stroke-related skeletal muscle changes: A review for the geriatric clinician. J. Geriatr. Phys. Ther..

[27] Hunter S.K., Pereira H.M., Keenan K.G. (2016). The aging neuromuscular system and motor performance. J. Appl. Physiol..

[28] Dimyan M., Cohen L. (2011). Neuroplasticity in the context of motor rehabilitation after stroke. Nat. Rev. Neurol..

[29] Yi Y., Shim J.S., Oh B.M., Seo H.G. (2017). Grip Strength on the Unaffected Side as an Independent Predictor of Functional Improvement After Stroke. Am. J. Phys. Med. Rehabil..

